# Requirement of a Blocking Step in Affinity Purification of Polyclonal Antibodies

**Published:** 2015

**Authors:** Sepideh Hamzehlou, Paul R. Albert, Mohammad M Farajollahi

**Affiliations:** 1 *Cellular and Molecular Research Center, Department of Medical Biotechnology, Faculty of Allied Medical Sciences, Iran University of Medical Sciences, Tehran, Iran.*; 2 *Ottawa Hospital Research Institute, Department of Medicine, Faculty of Medicine, University of Ottawa, Ottawa, Canada.*

**Sir,**

Antibodies play an essential role in medicine. Today diagnosis and treatment of many diseases require these “magic bullets”. Further-more, antibodies are essential components of a myriad of biomedical research. Hence, producing high quality premium antibodies is a great concern in molecular medicine and biotechnology. Since antibodies are produced in complex matrices *i.e.* serum for polyclonal antibody and cell culture media for monoclonal antibody, efficient purification of antibodies becomes imperative. Affinity purification is among the most extensively used methods for antibody purification. Affinity purification offers high selectivity and purity levels often above 95% in one step (1). Due to high specificity, antigen-specific affinity purification is the most popular method for affinity purification (2). Non-specific bindings (NSB) in affinity purification of antibodies are required to be minimized if antibodies are to achieve high analytical sensitivity and specificity. Various measures are taken to reduce NSB in affinity purifications. For instance, it is routine to use wash buffer containing additional salt or detergent to disrupt any weak interactions. Non-specific binding to protein A Sepharose and protein G Sepharose in insulin autoantibody assays may be reduced by pre-treatment with glycine or ethanolamine (3). A recent approach to minimizing non-specific protein interactions in high throughput screens has utilized pre-equilibration of affinity surfaces with thio-cyanate (4). In the present study, we examined whether blocking the affinity resin with bovine serum albumin (BSA) significantly reduces NSB in antigen-specific affinity purification of antibodies.

**Fig 1:**
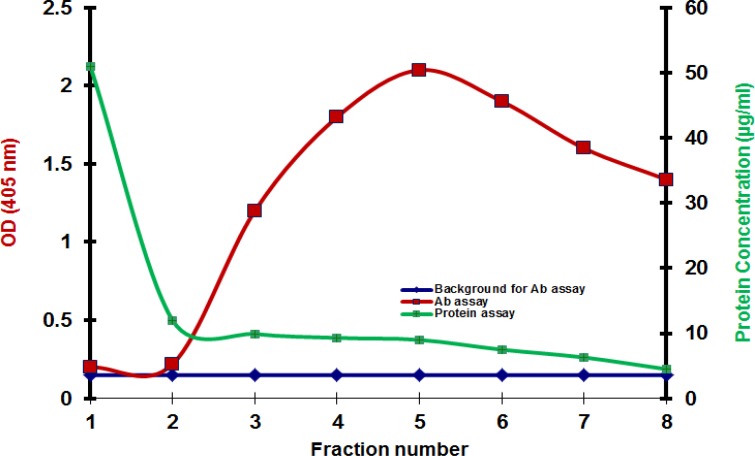
Results of immunoaffinity purification when the affinity beads are not blocked with BSA. Results of antibody assay and protein assay do not match. The left vertical scale shows ELISA results and the right vertical scale shows BCA results

**Fig 2:**
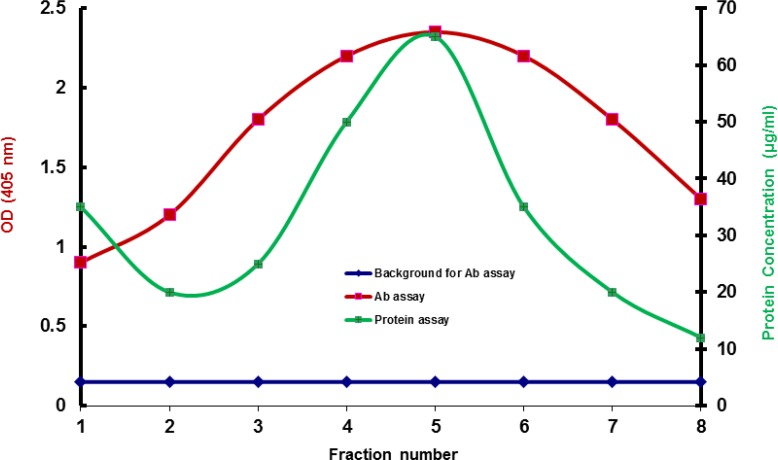
Results of immunoaffinity purification when the affinity beads are blocked with BSA. Results of antibody assay and protein assay properly matched. The left vertical scale shows ELISA results and the right vertical scale shows BCA results

TNFAIP8 peptide [tumor necrosis factor-alpha (TNFα)-induced protein 8] and rabbit serum containing anti-TNFAIP8 antibody both obtained from Sigma were used to conduct immunoaffinity purification in two methods: conventional and modified. Both immunoaffinity purifications were performed using NHS-Activated Agarose dry resin (Pierce, Rockford, IL, USA) according to the manufacturer’s protocol.The modified method, however, included an extra step, a blocking step with BSA, performed after quenching with 1 M ethanolamine. In the blocking step, a volume of 2 ml of 1% BSA in phosphate buffer saline (PBS) was applied to the column and mixed for 15 minutes and incubated for an additional 30 minutes without mixing at room temperature. Antibody concentrations and protein concentrations of the fractions eluted from the affinity column were then measured by ELISA and bicinchoninic acid (BCA) assay. We found that in conventional method, protein concentrations did not correspond to antibody concentrations and two graphs had totally different trends (figure 1). Protein concentrations corresponded to antibody concentrations only in the modified method (figure 2). 

We have already shown that in enzyme-linked immunosorbet assay (ELISA) the least NSB can be obtained with BSA compared to other blocking agents such as milk and casein (5). We believe that in the absence of the blocking agent, non-specific antibodies or proteins physically adsorb on the chromatography support surface through weak interactions and because physical adsorption is weaker compared to binding of the antibody to the specific antigen, non-specific antibodies if not removed after wash are likely to elute prior to specific antibodies in the initial fractions. Therefore, when the blocking step is not carried out, the purification process is error-prone: since it is common for the researchers to measure the protein levels of fractions and select the fractions with high protein levels**, **the correct purified antibody fractions would be missed and the wrong fractions would be saved for future assays rendering subsequent studies less efficient.To the best of our knowledge, there are no data about a “blocking” step in affinity purification of antibodies in the literature.
